# Supplementary material for: The institutional origins of vaccines distrust: Evidence from former-Soviet countries
Source: PLoS One. 2023 Mar 1;18(3):e0282420. doi: 10.1371/journal.pone.0282420 (PMC9977043; doi:10.1371/journal.pone.0282420)
Supplement: S1 Table — (PDF) [file pone.0282420.s001.pdf]

Table S1 The Descriptive Statistics of the WGM 2018 Research Sample.

	Count	Mean	St. Dev.	Min	Max
Trust in:					
Vaccine Efficiency	108275	4.378	0.967	1	5
Vaccine Safety	108142	4.246	1.088	1	5
Government health advice	115673	3.008	0.872	1	4
Medical advice from doctors	119827	3.247	0.768	1	4
Medical personnel	121799	3.204	0.822	1	4
Hospitals and health clinics	118854	0.753	0.431	0	1
Government	112645	2.558	1.051	1	4
People in neighbourhood	121222	3.008	0.900	1	4
Age	123789	42.606	18.134	15	99
Female	124349	0.542	0.498	0	1
Any exposure to Soviet communism	124349	0.151	0.358	0	1
Years under Soviet communism	124349	3.725	10.533	0	70

Source: Authors' own tabulation based on WGM 2018.
