# Supplementary material for: The institutional origins of vaccines distrust: Evidence from former-Soviet countries
Source: PLoS One. 2023 Mar 1;18(3):e0282420. doi: 10.1371/journal.pone.0282420 (PMC9977043; doi:10.1371/journal.pone.0282420)
Supplement: S2 Table — (PDF) [file pone.0282420.s002.pdf]

Table S2 The Dates of the Beginning and the End of the Exposure to Soviet Communism in Analysed Post-Communist Counties.

	Entry										
Exit	1917	1919	1920	1921	1922	1939	1940	1947	1948	1949	1952
1989								Romania		Poland	
1990				Georgia		Lithuania		Bulgaria	Czech Republic, Slovakia	Hungary	
1991		Belarus, Ukraine	Armenia, Azerbaijan		remaining USSR Republics		Latvia				
1992					Estonia						
1995	Russia										

Notes: Remaining USSR republics are: Kazakhstan, Kyrgyzstan, Moldova, Mongolia, Tajikistan, Turkmenistan, Uzbekistan. The total sample is comprised also of the following countries: Afghanistan, Albania, Algeria, Argentina, Australia, Austria, Bangladesh, Belgium, Benin, Bolivia, Botswana, Bosnia and Herzegovina, Brazil, Burundi, Cambodia, Cameroon, Canada, Chile, Colombia, Comoros, Costa Rica, Cyprus, Denmark, Dominican Republic, Ecuador, El Salvador, Eswatini, Ethiopia, Finland, France, Gabon, Gambia, Greece, Guatemala, Haiti, Honduras, Iceland, India, Indonesia, Iran, Ireland, Israel, Italy, Ivory Coast, Japan, Jordan, Kosovo, Kuwait, Kenya, Lebanon, Liberia, Luxembourg, Macedonia, Malawi, Malaysia, Malta, Mauritius, Mexico, Montenegro, Morocco, Mozambique, Myanmar, Namibia, Nepal, Netherlands, New Zealand, Niger, Nigeria, Northern Cyprus, Norway, Pakistan, Palestinian Territories, Panama, Paraguay, Peru, Philippines, Portugal, Rwanda, Saudi Arabia, Serbia, Singapore, South Africa, Spain, Sri Lanka, Sweden, Switzerland, Taiwan, Tanzania, Thailand, Togo, Turkey, Uganda, United Arab Emirates, United Kingdom, United States, Uruguay, Yemen, Zimbabwe.
Source: Authors' own tabulation based on dates of the socialist constitution and first free democratic elections. For the remaining USSR Republics, dates of 1922 and 1991 used as the beginning and end of communism.
