# Supplementary material for: The institutional origins of vaccines distrust: Evidence from former-Soviet countries
Source: PLoS One. 2023 Mar 1;18(3):e0282420. doi: 10.1371/journal.pone.0282420 (PMC9977043; doi:10.1371/journal.pone.0282420)
Supplement: S3 Table — (PDF) [file pone.0282420.s003.pdf]

Table S3 Effects of the Exposure to Soviet Communism on Trust in Vaccines' Efficiency and Safety.

	Vaccine efficiency	Vaccine efficiency
Exposure (continuous)	-0.038***	-0.040***
	(0.014)	(0.015)
N	107868	107745
R^2	0.100	0.143
Exposure (dummy)	-0.071**	-0.085**
	(0.027)	(0.036)
N	107868	107745
R^2	0.100	0.143
Age-fixed effect	Yes	Yes
Country-fixed effect	Yes	Yes
Country-specific time trend	Yes	Yes

Notes: The continuous measure of exposure is the length of exposure to Soviet communism in years smoothed with inverse hyperbolic sine function. The dummy measure of exposure is the having any exposure to Soviet communism. The continuous measure of exposure is the length of exposure to Soviet communism in years smoothed with inverse hyperbolic sine function. Standard errors clustered by country. Statistical significance: * – $p < 0.10$, ** – $p < 0.05$, *** – $p < 0.01$.
Source: WGM 2018
