# Supplementary material for: The institutional origins of vaccines distrust: Evidence from former-Soviet countries
Source: PLoS One. 2023 Mar 1;18(3):e0282420. doi: 10.1371/journal.pone.0282420 (PMC9977043; doi:10.1371/journal.pone.0282420)
Supplement: S4 Table — (PDF) [file pone.0282420.s004.pdf]

Table S4 Effects of the Exposure to Soviet Communism on Generalized Trust and Trust in Medical Care.

	Government Health Advice	Medical Advice from Doctors	Medical Personnel	Hospital and Health Clinics	Government	People in Neighbourhood
Exposure (continuous)	-0.048***	-0.037***	-0.041***	-0.013***	-0.057***	-0.022**
	(0.013)	(0.009)	(0.010)	(0.005)	(0.015)	(0.011)
N	115213	119351	121297	118357	112221	120725
R^2	0.146	0.126	0.139	0.099	0.209	0.140
Exposure (dummy)	-0.079***	-0.056***	-0.063***	-0.015	-0.096***	-0.041**
	(0.028)	(0.018)	(0.021)	(0.012)	(0.032)	(0.021)
N	115213	119351	121297	118357	112221	120725
R^2	0.145	0.126	0.139	0.099	0.209	0.140
Age-fixed effect	Yes	Yes	Yes	Yes	Yes	Yes
Country-fixed effect	Yes	Yes	Yes	Yes	Yes	Yes
Country-specific time trend	Yes	Yes	Yes	Yes	Yes	Yes

Notes: The continuous measure of exposure is the length of exposure to Soviet communism in years smoothed with inverse hyperbolic sine function. The dummy measure of exposure is the having any exposure to Soviet communism. The continuous measure of exposure is the length of exposure to Soviet communism in years smoothed with inverse hyperbolic sine function. Standard errors clustered by country. Statistical significance: * – $p < 0.10$, ** – $p < 0.05$, *** – $p < 0.01$.
Source: WGM 2018
