# Supplementary material for: The institutional origins of vaccines distrust: Evidence from former-Soviet countries
Source: PLoS One. 2023 Mar 1;18(3):e0282420. doi: 10.1371/journal.pone.0282420 (PMC9977043; doi:10.1371/journal.pone.0282420)
Supplement: S5 Table — (PDF) [file pone.0282420.s005.pdf]

Table S5 Robustness of the Main Results to Orthodox Country

	Vaccine efficiency	Vaccine efficiency
Exposure (continuous) × Orthodox	-0.044	0.041
	(0.028)	(0.030)
N	11186	11151
R^2	0.065	0.078
Exposure (dummy) × Orthodox	-0.083	0.048
	(0.062)	(0.077)
N	11186	11151
R^2	0.065	0.078
Age-fixed effect	Yes	Yes
Country-fixed effect	Yes	Yes
Country-specific time trend	Yes	Yes

Notes: The treatment variable is the length of exposure to Soviet communism in years smoothed with inverse hyperbolic sine function. The countries classified as predominantly Orthodox Christian are: Belarus, Bulgaria, Cyprus, Georgia, Greece, Macedonia, Moldova, Montenegro, Romania, Russia, Serbia, Ukraine. Standard errors clustered by country. Statistical significance: * – $p < 0.10$, ** – $p < 0.05$, *** – $p < 0.01$.
Source: WGM 2018.
