# Supplementary material for: The institutional origins of vaccines distrust: Evidence from former-Soviet countries
Source: PLoS One. 2023 Mar 1;18(3):e0282420. doi: 10.1371/journal.pone.0282420 (PMC9977043; doi:10.1371/journal.pone.0282420)
Supplement: S1 Fig — (PDF) [file pone.0282420.s006.pdf]

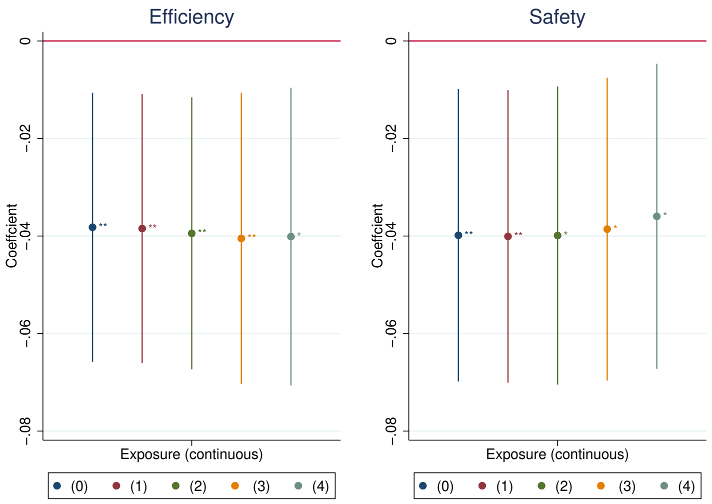

Fig. S1 Robustness of the Main Results to Additional Controls

Notes: The treatment variable is the length of exposure to Soviet communism in years smoothed with inverse hyperbolic sine function. Control variables depending on the model specification: (0)-(4) age fixed-effects, gender, country fixed-effects and country-specific time trend, and (1)-(4) dummy for urban or rural area, (2)-(4) having children, (3)-(4) dummy for individual religiosity, and (4) education level (primary, secondary, tertiary). Presented confidence intervals at 95% significance level are obtained using standard errors clustered by country. Statistical significance: * – $p < 0.10$, ** – $p < 0.05$, *** – $p < 0.01$.
Source: WGM 2018.
