# Supplementary material for: The institutional origins of vaccines distrust: Evidence from former-Soviet countries
Source: PLoS One. 2023 Mar 1;18(3):e0282420. doi: 10.1371/journal.pone.0282420 (PMC9977043; doi:10.1371/journal.pone.0282420)
Supplement: S1 Checklist — This PDF file contains STROBE check-list for analysis using cross-section data. (PDF) [file pone.0282420.s007.pdf]

STROBE Statement—Checklist of items that should be included in reports of ***cross-sectional studies***

	Item No.	Recommendation	Page No.	Relevant text from manuscript
**Title and abstract**	1	(*a*) Indicate the study's design with a commonly used term in the title or the abstract	1	
		(*b*) Provide in the abstract an informative and balanced summary of what was done and what was found	1	
**Introduction**				
Background/rationale	2	Explain the scientific background and rationale for the investigation being reported	1-4	Background: Lines 10-19<br>Rationale: Lines 33-71
Objectives	3	State specific objectives, including any prespecified hypotheses	2	Lines 19-20
**Methods**				
Study design	4	Present key elements of study design early in the paper	2	Lines 21-32
Setting	5	Describe the setting, locations, and relevant dates, including periods of recruitment, exposure, follow-up, and data collection	4	Lines 75-78
Participants	6	(*a*) Give the eligibility criteria, and the sources and methods of selection of participants	4	Line 77
Variables	7	Clearly define all outcomes, exposures, predictors, potential confounders, and effect modifiers. Give diagnostic criteria, if applicable	4-5	Outcomes: Lines 80-98<br>Exposures: Lines 100-110
Data sources/ measurement	8*	For each variable of interest, give sources of data and details of methods of assessment (measurement). Describe comparability of assessment methods if there is more than one group	4-5	Trust in vaccines: Lines 83-86<br>Trust in institutions: Lines 91-98<br>Exposure: 106-110
Bias	9	Describe any efforts to address potential sources of bias	5<br>7	Lines 110-115<br>Lines 155-163
Study size	10	Explain how the study size was arrived at	5	Lines 113-115
Quantitative variables	11	Explain how quantitative variables were handled in the analyses. If applicable, describe which groupings were chosen and why	4	Lines 85-86
Statistical methods	12	(*a*) Describe all statistical methods, including those used to control for confounding	5-6	Lines 116-129
		(*b*) Describe any methods used to examine subgroups and interactions	8	Lines 164-170
		(*c*) Explain how missing data were addressed		Not applicable
		(*d*) If applicable, describe analytical methods taking account of sampling strategy	6	Lines 127-129
		(*e*) Describe any sensitivity analyses	8	Lines 170-172

**Results**				
Participants	13*	(a) Report numbers of individuals at each stage of study—eg numbers potentially eligible, examined for eligibility, confirmed eligible, included in the study, completing follow-up, and analysed		Not applicable
		(b) Give reasons for non-participation at each stage		Not applicable
		(c) Consider use of a flow diagram		Not applicable
Descriptive data	14*	(a) Give characteristics of study participants (eg demographic, clinical, social) and information on exposures and potential confounders	5	Lines 99-104
		(b) Indicate number of participants with missing data for each variable of interest	SI	Table S1
Outcome data	15*	Report numbers of outcome events or summary measures	SI	Table S1
Main results	16	(*a*) Give unadjusted estimates and, if applicable, confounder-adjusted estimates and their precision (eg, 95% confidence interval). Make clear which confounders were adjusted for and why they were included	6	Unadjusted: Lines 132-137<br>Adjusted: Lines 138-154
		(*b*) Report category boundaries when continuous variables were categorized		Fig 1
		(*c*) If relevant, consider translating estimates of relative risk into absolute risk for a meaningful time period		Not applicable
Other analyses	17	Report other analyses done—eg analyses of subgroups and interactions, and sensitivity analyses	8<br>SI	Fig 5<br>Fig S1
**Discussion**				
Key results	18	Summarise key results with reference to study objectives	9	Lines 194-199
Limitations	19	Discuss limitations of the study, taking into account sources of potential bias or imprecision. Discuss both direction and magnitude of any potential bias	7	Lines 155-160
Interpretation	20	Give a cautious overall interpretation of results considering objectives, limitations, multiplicity of analyses, results from similar studies, and other relevant evidence	9	Lines 187-206
Generalisability	21	Discuss the generalisability (external validity) of the study results	7	Lines 160-163
**Other information**				
Funding	22	Give the source of funding and the role of the funders for the present study and, if applicable, for the original study on which the present article is based	10	Lines 219-223

*Give information separately for exposed and unexposed groups.

Note: An Explanation and Elaboration article discusses each checklist item and gives methodological background and published examples of transparent reporting. The STROBE checklist is best used in conjunction with this article (freely available on the Web sites of PLoS Medicine at http://www.plosmedicine.org/, Annals of Internal Medicine at http://www.annals.org/, and Epidemiology at http://www.epidem.com/). Information on the STROBE Initiative is available at www.strobe-statement.org.
