# Supplementary material for: The institutional origins of vaccines distrust: Evidence from former-Soviet countries
Source: PLoS One. 2023 Mar 1;18(3):e0282420. doi: 10.1371/journal.pone.0282420 (PMC9977043; doi:10.1371/journal.pone.0282420)
Supplement: Attachment — Submitted filename: Response to Reviewers.pdf [file pone.0282420.s008.pdf]

Report to the referees

Reviewer #1: In the current manuscript, entitled "The institutional origins of vaccines distrust: Evidence from former-communist countries", the authors show the association between being exposed to Soviet communism affect trust in vaccines, which is explained by weaker institutional and governmental trust. I enjoyed reading the **manuscript as it was clearly written**, and deals with what I think is an extremely important topic, namely, vaccine hesitancy. However, I believe that there are two major issues that should be addressed. I list these below, and I hope that the author would find them useful.

1. I think that some more elaboration on why exposure to Soviet communism would be relevant to vaccine hesitancy is warranted. Is it something about the history of those countries? Is it something about the ideology? Culture? For example, recent research by Maaravi and colleagues shows the relationship between individualism / collectivism and COVID-19 spread (which I can only assume is correlated with exposure to Soviet communism, although they show a trend that to some extent is opposite to the current research results). Other unpublished research by Adam-Troian and colleagues (see here https://psyarxiv.com/nzg7x) examines the association between collectivism, masculinity (controlling for other relevant variables such as Human Development Index) to belief in conspiracy theories (which was linked to institutional trust and vaccine hesitancy). All of this to say that I think that some more precision about what is it exactly in exposure to Soviet communism that leads to vaccine hesitancy and lower levels of institutional and governmental trust is important.

R: Thank you for pointing out different possible mechanisms explaining our findings and the references. We have revised the two suggestions and expanded the introduction on pages 3-4 as well as the discussion on page 10. We link the exposure to Soviet communism effects to the experiences of enforced vaccinations common to all countries in the Soviet bloc, however we are unable to test nor rule out conclusively other potential mechanisms such as collectivist, individualistic or masculinity values.

2. I believe that in order for the analysis to be persuasive it has to control for other relevant variables that are related to institutional, governmental and vaccine trust (both on the country and individual levels). Both the work by Adam-Troian et al. and Maaravi et al. I mentioned above control for some relevant such measures.

R: Thank you very much for the very relevant references and methodological suggestion. We have cited appropriately both studies in pages 3 and 10 and linked their results to our conclusions. Regarding the use of the control variables they use, please note our specification includes both country fixed-effects, which accounts for both country level time-invariant observable and unobservable differences. The inclusion of country level control variables as in the studies mentioned would create in our setting a problem of perfect multicolinearity. We included also additional individual controls to find robust results reported in Figure A1 of the Appendix. We believe however that since the measure of exposure to communism is exogenous (determined by year and place of birth) the inclusion of individual level attitudes or beliefs could lead to a problem of "bad control" as defined in Angrist and Pischke (2008). Hence, the former robustness check of various country sub-samples is better suited to our analysis and as such we place it in the main body of the paper.

Angrist, Joshua D., and Jörn-Steffen Pischke. *Mostly harmless econometrics*. Princeton University Press, 2008.

3. The results on “psychological mechanisms” go some way to address my first comment, but I think that there’s still a need to further explain what is it in the exposure to Soviet communism that drives these effects. Is it something about the type of regime? Is it something about the process in which these regimes collapsed and what happened since that broke individuals’ trust? Is the trend only relevant to Soviet communism? Why would that be the case? Is it because it collapsed? Is it just because vaccination was obligatory in the former Soviet Union? In other words, I think that more is needed to be done in order to explain whether the trends the authors report in the paper are specific to Soviet communism, and if so, why? Or whether it is something that has to do with regime changes or authoritarian regimes, etc.

R: We have now provided a more extensive and hopefully convincing explanation both in the Introduction and discussion sections referring to the general lack of transparency in public institutions, the power of Soviet authorities over individuals as well as military-like enforcement of vaccinations. The conspiracy and misinformation related to health care systems in Soviet times are associated not only with corruption but also with Soviet collectivism that paid little attention to individuals as well as with a denial of any danger in severe cases of environmental pollution, including radioactive contamination, as for example within first days after the accident in Chernobyl nuclear power plant.

4. As a minor comment, I think that it would be good to label the y-axis in the figures. I am not sure it is clear.

R: Thank you for this comment, we included it in our Figures which makes them much easier to read.

5. As another minor comment, maybe some more explanation about how exposure to communism was computed in the model. The authors indicate that it was measured in two different ways, but then it was less clear to me how these two different ways were combined.

R: Amended by providing on page 6 the general rule of setting the date of communism start to the date of Soviet constitution and of its end to the date of first free parliamentary (or presidential in the case of Russia) elections, and more details (specific dates separately for each country) in the Appendix.

Reviewer #2: When I saw the Abstract for this paper, I was very excited. I undertake sociological research on 'trust in vaccinations', so to see a potential paper exploring the impact of communism on trust in vaccinations was exciting. The authors use existing data from the Wellcome Global Monitor (which is fine) and undertake some statistical analysis (which I am not equipped to review). The Introduction is too broad, and does not adequately present and critique global literature on 'trust in vaccinations' and needs to engage much more deeply with the sociology of trust. There is a huge literature on trust in governments, medical institutions, pharmaceutical industries etc - very little if any of this has been engaged with. The authors need to be able to set their study and findings within these literatures - so they can identify 'what we already know' and 'what's new'. On lines 24-

26, there's a referencing problem which goes throughout the paper (the first references are 6 and 1? In the Discussion, the authors could/should engage with sociologists who have explored how trust differs between different societies - for example, Fukuyama explored trust in different societal structures. In the Discussion, the paragraph on lines 151-161 does not seem to be relevant at all - the authors have introduced COVID-19 context, but the Wellcome data predated COVID-19.

R: We have expanded the literature on government trust in more detail and refer to the discussion in sociology. In addition to Simmel's and Fukuyama's seminal work on trust, we refer to more recent research on trust and confidence in public institutions in post-communist and other countries.

Thank you for your point on the pre-COVID data. We explained why the attitudes towards vaccination in general might be relevant for the COVID-19 vaccine hesitancy on page 3. We have dealt with the referencing problem as well.

Reviewer #3: Title: The institutional origin of vaccine distrust: Evidence from former-communist countries

Journal: Plos One

The article is a study of vaccine distrust in former-communist countries. Based on an analysis of data from the Welcome Global Monitor (WGM) database, the authors seek to show that exposure to communist regimes play an important role in distrust in public institutions that leads to vaccine hesitancy. Considering the scale of the challenge associated with vaccine hesitancy, especially during a pandemic, this article's contributions to policy framing and guidelines are timely and necessary.

While the article does offer an important and timely exploration of vaccine hesitancy in former-communist countries, there is still some work to be done in establishing a coherent narrative, and tightening the arguments by addressing not only the results of the analysis but also more of the literature on trust, hesitancy and health policy. Addressing these issues (and a few other minor comments) will strengthen the arguments made in the article. More specific comments follow:

1. The authors provide very clear and admirable inclusion/exclusion criteria for which they should be congratulated. However, the grouping of former-communist counties and the assumption that all communist experiences are similar might be problematic. While it might be difficult to account for the broad variety of experiences based on the available database, I suggest the authors address this methodological difficulty.

R: We have done a number robustness check looking at the different types of communism, namely Soviet Union members, Russia, and selected Soviet Republics with strongest links to Western Europe, as presented in Subsection 3.3 Robustness on page 9.

2. On page 3, the authors claim that the "military-like organizations and its compulsory participation lead to a questioning of such campaigns after transition". While these claims might be intuitive to

scholars of former-communist health systems, many of the journal's readers may not be acquainted with this argument. Therefore, it would be helpful to add sources baking the claim.

R: Thank you for noting that, we have added additional references to the literature that documents these Soviet-specific practices.

3. In the Data section (page 4) the authors address the length of the influence of life under communist regimes. If possible, it would be helpful to address inter-generational effects as well.

R: We have discussed the role of intergenerational transmission of preferences, which is likely to take place as far as trust is concerned. However, if the preferences were not transmitted over generations and individuals unexposed to communism (or exposed to communism for relatively short period of time if we examine extensive margin of the exposure) in the formerly communist countries did not inherit any attitudes towards vaccination nor mistrust from their parents exposed to communism (for relatively long periods of time), the estimated effects of the exposure to communism should be even stronger. Therefore, the intergenerational transmission is not a problem in the study as it would result to the in the underestimation of the effects of the exposure to communism.

The fact that we find an effect on the intensive margin and not only on the extensive margin of the exposure suggests that documented effects are not driven by the transition but indeed by the exposure to communism. Despite our results support the trust mechanism, but we cannot conclusively rule out other mechanisms or say whether other authoritarian regimes or forms of communism would have a similar effect. Despite Soviet communism as one of authoritarian regimes shares a number of similarities with other communist regimes (such as for example forced vaccination in China), remains distinct from other authoritarian regimes. Our results are consistent with trust playing an important role, but we cannot test in the present study whether other forms of authoritarian regimes or forms of communism could have the same effect.

4. In their analysis of Underlying mechanisms (page 6) the authors claim that trust has deteriorated. This framing assumes that trust existed in the past, however it's unclear in the text if this is indeed the case.

R: We have clarified this point and elaborated more on the underlying mechanisms in Subsection 3.2.

5. The article has the potential to contribute greatly to the discussion on trust in state institutions and vaccine hesitancy. However, in order to do so more attention is needed to the already broad body of knowledge on the issue. The theorizing of trust and hesitancy appears only in the discussion, and it would be helpful to address it earlier in the article.

R: We have added more on trust and vaccine hesitancy, and especially more theory in the earlier parts of the paper.

6. Along the same line, a clearly framed discussion on trust in institutions and hesitancy would help the authors take their place in a very active field (and explain how is this case different from other cases).

R: We have improved the discussion.

7. I suggest the authors look at Heidi Larson's book Stuck (2020) on vaccine rumours. While other works by Larson are cited, this might be an interesting read in terms of framing the context.

R: Thank you, we have included it.

8. On page 8, the authors shift to COVID-19. While this is obviously a burning issue (and of relevance to the article) it is not necessarily clear how a study conducted pre-COVID-19 relates to the matter at hand. A clearer connection should be made in order for this important section to be a better fit.

R: We have added references showing that hesitancy to other vaccines correlated with COVID-19 too.

9. The authors end with a very interesting note on the use of compulsory mechanisms. I encourage the authors to be even bolder in their conclusions, and odd their recommendations.

R: We have sharpened our conclusions, thank you for encouraging us to do so.

10. As someone who is interested in trust in the state and health policy I have truly enjoyed reading this article. I thank the authors for their analysis.

R: Many thanks for reading our paper, we feel very honoured with your remark.
