# Supplementary material for: The institutional origins of vaccines distrust: Evidence from former-Soviet countries
Source: PLoS One. 2023 Mar 1;18(3):e0282420. doi: 10.1371/journal.pone.0282420 (PMC9977043; doi:10.1371/journal.pone.0282420)
Supplement: Attachment — Submitted filename: Response to Reviewers.pdf [file pone.0282420.s009.pdf]

Response to the reviews

In the abstract it states "… from a long list of world countries…", but I think there is no need to be so vague. I'd recommend stating the exact number. Also in the abstract, there is causal language without any causal tests to justify it: "past exposure to Soviet communism reduces trust in vaccinations". I think based on the correlational nature of the data, a more accurate statement would be something like "past exposure to Soviet communism is associated with reduced trust in vaccinations". I would tone back any causal language that occurs throughout the manuscript to be more in line with the nature of the data.

Thank you for this important comment, we have amended the language to be more precise in terms of the nature of our findings throughput the entire article. We also provided the exact number of examined countries (122) in the Abstract and in the Data description (page 5).

In the introduction, it was mentioned that experiencing mass vaccinations in Soviet countries might lead to vaccine distrust. This is perhaps true, but voluntary vaccination rates for the flu are considerably higher in former communist East Germany compared to West Germany (Rehmet et al., 2002), which might suggest it is, at least, more complicated.

We discuss it the revised draft referring to the evidence suggesting difference between East and West Germany in Rehmet et al. (2002) (page 10). A stated in the paper, the difference arises due to a higher access to family physicians in the East Germany, which are mainly the ones making vaccinations recommendations, given that there is no need of GP referral in Germany to see a specialist. Moreover, we include the ECDC evidence for seasonal flu suggesting that, as we argue, eastern European countries exhibit the lowest rates of influenza vaccination among olde people and pregnant women (ECDC, 2023): https://www.ecdc.europa.eu/en/publications-data/seasonal-influenza-vaccination-antiviral-use-eu-eea-member-states.

Based on the argument presented in the manuscript, I was partly expecting a mediational analysis of the effect of communism on vaccination hesitancy. Namely, that it is mediated by lack of (some form of) trust. If such an analysis isn't done, then talks about mechanisms should be more speculative. Recently published work that found trust in government mediated the association between former communist federal states and COVID-19 vaccination rates within Germany (at the state level rather than individual level; Martens, 2022). This is a highly relevant article for the current work.

Thanks for the reference. We now cite Martens (2022) in the updated version of the manuscript when discussing potential hypotheses that might be explaining the association in the Introduction (page 5).

Regarding the evidence on mechanisms, I believe this is probably a confusion by the different use of the word across disciplines. Typically, the analysis of mechanisms in economic papers is based on heterogeneous effects or evidence supporting a particular hypothesis, rather than conducting mediational analysis, which is a technique rarely used in our discipline. As the reviewer states, in our paper, our section on mechanism shows that Soviet communism reduced trust in these institutions, which is consistent with the hypothesis that trust could be driving the negative association between exposure to communism and trust in vaccine effectiveness and safety.

We understand however the section might create confusion to colleagues of other disciplines and therefore we have toned down the results of the section and avoid the use of the term analysis of mechanism when presenting the results on trust. We hope the section is better communicated now.

I believe the results are presented in a more confusing manner than they need be, and they generally lack detail. What analyses were specifically run is not clearly presented. I assume these are a series of regressions, but then what are the exact results (e.g., B, CI, etc.)? I see figures, but tables with specific values would provide more information and aid in reproducing the effects and in any metanalysis down the road, etc. Consequently, although I generally like what the manuscript attempts to do, I feel unable to fully judge it because I am not confident in precisely what analyses were run or what was found.

Thank you for this comment, we have revised the description of the methods in the Identification Strategy section (page 7) to make it clearer. We run two separate OLS estimations for the trust in vaccines' efficiency and safety, using either dichotomous or continuous measure of exposure to communism as the explanatory variable, controlling for fixed effects of birth year, country of birth as well as for the country-specific time trends. In addition, we control for gender and a number of other controls in the robustness tests.

We have chosen to present the results in the graphic form so that they are more communicative. The point estimates are shown along with the Confidence Intervals at 95% significance level, and the statistical significance of the estimate is shown using asterisks by the point estimate. The same amount of information presented in a table would be less readable, thus we decided to stay with the Figures in the main body of the paper and provide all the details in the following Tables added to the appendix. We have also revised the Notes to all Figures to make sure all these information are a part of each Figure.

Table S3 Effects of the Exposure to Soviet Communism on Trust in Vaccines' Efficiency and Safety.

	Vaccine efficiency	Vaccine efficiency
Exposure (continuous)	-0.03820***	-0.03984***
	(0.0139)	(0.0152)
N	107868	107745
R2	0.100	0.143
Exposure (dummy)	-0.07132**	-0.08504**
	(0.0273)	(0.0357)
N	107868	107745
R2	0.100	0.143
Age-fixed effect	Yes	Yes
Country-fixed effect	Yes	Yes
Country-specific time trend	Yes	Yes

Notes: The continuous measure of exposure is the length of exposure to Soviet communism in years smoothed with inverse hyperbolic sine function. The dummy measure of exposure is the having any exposure to Soviet communism. The continuous measure of exposure is the length of exposure to Soviet communism in years smoothed with inverse hyperbolic sine function. Standard errors clustered by country. Statistical significance: * – p < 0.10, ** – p < 0.05, *** – p < 0.01.
Source: WGM 2018

Table S4 Effects of the Exposure to Soviet Communism on Generalized Trust and Trust in Medical Care.

	Government Health Advice	Medical Advice from Doctors	Medical Personnel	Hospital and Health Clinics	Government	People in Neighbourhood
Exposure (continuous)	-0.04838***	-0.03662***	-0.04108***	-0.01307***	-0.05669***	-0.02178**
	(0.0128)	(0.0095)	(0.0097)	(0.0049)	(0.0154)	(0.0106)
N	115213	119351	121297	118357	112221	120725
R2	0.146	0.126	0.139	0.099	0.209	0.140
Exposure (dummy)	-0.07932***	-0.05639***	-0.06273***	-0.01480	-0.09586***	-0.04130**
	(0.0282)	(0.0185)	(0.0206)	(0.0117)	(0.0319)	(0.0208)
N	115213	119351	121297	118357	112221	120725
R2	0.145	0.126	0.139	0.099	0.209	0.140
Age-fixed effect	Yes	Yes	Yes	Yes	Yes	Yes
Country-fixed effect	Yes	Yes	Yes	Yes	Yes	Yes
Country-specific time trend	Yes	Yes	Yes	Yes	Yes	Yes

Notes: The continuous measure of exposure is the length of exposure to Soviet communism in years smoothed with inverse hyperbolic sine function. The dummy measure of exposure is the having any exposure to Soviet communism. The continuous measure of exposure is the length of exposure to Soviet communism in years smoothed with inverse hyperbolic sine function. Standard errors clustered by country. Statistical significance: * – $p < 0.10$, ** – $p < 0.05$, *** – $p < 0.01$.
Source: WGM 2018

I think there should be more mention of religion or spirituality since we know it influences vaccination skepticism (e.g., Rutjens et al., 2021) as well as COVID-19 vaccination rates (Martens & Rutjens, 2022). Given that the Soviet Bloc is predominantly Orthodox Christian, how can we be sure this isn't a religion effect rather than a Soviet communism effect? I don't actually think it is since Martens (2022) found a communist effect in Asia, but I believe this should at least be addressed in text as it is a likely contributing factor to vaccination hesitancy. Perhaps as a future direction.

Thanks for the references. We have added to the introduction a reference to religion or spirituality as factors that may also affect vaccinations rate and cite the mentioned studies. Regarding whether the effect could be driven by Orthodox Christian, the use of country specific fixed effects lowers this concern. Moreover, we control for individual religiosity in the robustness checks to find reassuring results. Nonetheless, we test this hypothesis in the updated version of the manuscript (Figure S2) through exploring whether the association is stronger across Orthodox and non-Orthodox Soviet countries. We do not find any significant differences, as the coefficients on the interaction of the exposures (both continuous and dichotomous) with Orthodox country shown in the Table below are statistically indistinguishable from zero.

Table R1 Robustness to the Orthodox Christian Country.

	Vaccine efficiency	Vaccine efficiency
Exposure (continuous) × Orthodox	-0.04404	0.04090
	(0.0280)	(0.0304)
N	11186	11151
R2	0.065	0.078

Exposure (dummy) × Orthodox	-0.08267	0.04828
	(0.0619)	(0.0772)
N	11186	11151
R2	0.065	0.078
Age-fixed effect	Yes	Yes
Country-fixed effect	Yes	Yes
Country-specific time trend	Yes	Yes

Notes: The countries classified as predominantly Orthodox Christian are: Belarus, Bulgaria, Cyprus, Georgia, Greece, Macedonia, Moldova, Montenegro, Romania, Russia, Serbia, Ukraine. The continuous measure of Exposure to communism is the length of exposure to Soviet communism in years smoothed with inverse hyperbolic sine function. Standard errors clustered by country. Statistical significance: * – $p < 0.10$, ** – $p < 0.05$, *** – $p < 0.01$.
Source: WGM 2018.

What are the hypotheses precisely? In the introduction (sentences 5-9) it states that the paper examines the reaction to historical events (mass vaccination during Soviet times), weak trust in government and health system, suspicion of large business organizations (big pharma), and a reaction to egalitarian values of Soviet communism. Presumably how all of those are related to vaccine hesitancy (or vaccine trust as it is sometimes put). At other times the manuscript states it inquires about the effects of exposure to Soviet communism on trust in vaccines efficiency and safety (21-22), and whether communism explains different dimensions of vaccine trust (23-24). The first results presented give a breakdown of vaccine safety and efficiency (via a table) with some mention of communism.

Thanks for this comment. We have clarified it in the article by rephrasing the sentences 5-9 in the introduction and providing explicit hypothesis on page 4. Our hypothesis is that exposure to communism is associated vaccine scepticism i.e. reduced trust both in vaccines' safety and efficiency. The rationale for the hypothesis are the characteristics of the exposure to communism and experiences it entailed, specifically (forced) mass vaccination, generalized trust deterioration, reduced confidence in public institutions, health care system rooted in the Semashko model, prevalence of egalitarian values as well as suspicion of large business organization. These are the traits documented in the literature discussed and cited in the Introduction.

It next presents data to suggest an underlying mechanism (trust in government, doctors, etc.). From what I can tell, the section on underlying mechanisms is not actually testing underlying mechanisms, but rather an association consistent with the mechanism. Perhaps I've misinterpreted these results, but I believe that is partly part of the problem, since what was done is not clearly communicated

We believe this is probably a confusion by the different use of the word across disciplines. Typically, the analysis of mechanisms in economic papers is based on heterogeneous effects or evidence supporting a particular hypothesis, rather than conducting mediational analysis, which is a technique rarely used in our discipline. As the reviewer states, in our paper, our section on mechanism shows that Soviet communism reduced trust in these institutions, which is consistent with the hypothesis that trust could be driving the negative association between exposure to communism and trust in vaccine effectiveness and safety.

We understand however the section might create confusion to colleagues of other disciplines and therefore we have toned down the results of the section and avoid the use of the term analysis of mechanism when presenting the results on trust. We hope the section is better communicated now.

In addition, the distinction between intensive and extensive margin effects is interesting, but similar to above, it is difficult to follow what was precisely done here.

Thanks for the comment. Intensive and extensive margins are common terms in economics. In this case, the extensive margin of exposure to communist is a variable that takes the value of 1 if the individual is exposed to communism and 0 otherwise. The intensive margin measures the number of years of exposure of the individual to Soviet communism.

We understand PLOSONE is an interdisciplinary journal and therefore, we have replaced these terms in the updated version of the paper. Rather than using extensive and intensive margin of exposure to communism we have used the terms “exposed to communism (0-1)” and “number of years one was exposed to communism (0-70 years)”.

The last part of the results is on robustness of the association. I see no data or results measuring mass vaccinations during Soviet times, suspicion about large businesses, or egalitarian values, so contrary to the introduction, these were not evaluated. They might be aspects that support your hypotheses (which I take to be that Soviet communism in particular leads to lack of trust in a variety of areas, which then leads to vaccine hesitancy), but they aren't part of the data collection or analyses.

Thanks for this point, we have now stated the hypothesis and clarified what can be tested empirically, based on the available data. We are able to test a number of channels through which communism might have affected (e.g. Trust in medical advice from doctors, in medical personnel, government, etc.). We recognize that there are other potential attitudes we do not measure such as ‘suspicion about large businesses’, or ‘egalitarian values’ documented in the literature. In the revised article we make clear distinction between findings based on our own empirical analysis and where we rely on existing published evidence.

Generally speaking, I found the introduction and discussion to not be overly developed. I think sub-headings with clear points (perhaps framed around hypotheses) could help here. That might also help the results section (i.e., frame results more explicitly around hypotheses testing).

Thanks for this suggestion! We have expanded the discussion and organised the results and discussion with subheadings now.

It isn't clear to me why the focus is solely on Soviet communism. Is it because of mass vaccination efforts in Soviet communism countries that didn't happen in Asian/African communist countries? Asian communism is associated with lower vaccinations rates for COVID-19 (Martens, 2022), so perhaps it isn't something unique to Soviet communism. If it is something unique to Soviet communism then you would expect an effect with Soviet communism but not other forms of communism, but you can test this by including other forms of communism in the analyses. If running this test isn't possible for some reason, then I'd like to see more of an expansion on justifying why Soviet regions are the focus.

Thanks for the comment, we clarify that in the paper on page 5. We focused on Soviet communism for both practical and theoretical reasons. Firstly, for Soviet countries we could identify a relatively common treatment and transition period, as well as a homogeneous sample of what is a communist country. The inclusion of countries such as China or Vietnam, formally communist countries but with economic systems that have transitioned into capitalism on many aspects, would complicate the interpretation of the treatment effects. Similarly, the inclusion of some African and Asian countries that experienced for shorter periods different forms of communism (typically associated with civil

wars) and transitioned into right-wing dictatorships could difficult the interpretation of the results. Thus, through restricting the analysis to Soviet countries, which experienced a relatively common political and economic system with clear dates for the beginning and end of communism, and then, similar economic and political systems during transition, we could yield more homogeneous and easier to interpret results. Secondly, gathering precise and comprehensive information on dates of the beginning and end of communism in every world country that experienced communism would require resources that we do not have at the moment. Finally, it is worth mentioning we have mentioned this point in the text now.

Trust in vaccinations seems to be operationalized as both vaccine efficiency and safety. I'm not convinced that those are two measures of vaccine trust. Maybe they do (which would be good to hear justification for), but someone might have complete trust in taking a vaccine but think it is not efficient (e.g., it takes multiple injections for full immunity). Safety appears to be more directly about trust than efficiency, but neither are the most direct, which would be something like "I trust vaccines", which unfortunately doesn't appear to be part of the dataset. I'd recommend rethinking the label (hesitancy was used earlier in the paper, so might be used for consistency, but views/opinion could also work here. Skepticism is another possibility, and previous work that used the same data set called those variables skepticism; Martens & Rutjens, 2022) and/or justifying how those are actual measures of trust.

Thanks for this helpful suggestion. We have revised the article accordingly labelling examined beliefs as vaccine scepticism, and clearly state that we operationalize it by the two measures: of trust in vaccines efficiency and safety. We have expanded the points regarding vaccine hesitancy and scepticism of this in the paper, and more specifically mentioned that they refer to instrumental dimensions of trust which have been reported as the key variable in a number of studies.

I assume those two measures are highly correlated, which would be good to see since they are the main DVs. It might make sense to combine them into a scale which might help with any odd effects of any individual item, unless the authors are trying to make an argument more specifically about each aspect (efficiency and safety). I take it that this is more about hesitancy, so a scale might make sense here.

Thank you for this point. We have revised the text making sure that the two outcomes are interpreted separately, and the distinction between them is pronounced enough.

We have discussed the selection of the dependent variable at the very start of the research, and considered generating an index or a scale. We concluded these considerations with the decision to use the variables directly reported directly by the respondents for two main reasons.

First, we are not experts in the development of scales or indexes, but we are aware of the methodological complexities related to the development of an accurate and robust measures combining a number of variables, and one of them is free from limitations. We decided that each method has limitations reducing the conclusiveness of our empirical analysis in comparison to the simple analysis that we used as our main result.

Second, the interpretation of results using the exact questions respondents were asked in the survey is straightforward and accurate without any additional assumptions. As economists, we are hesitant to use any variable that is not directly observed in the data as the dependent variable, because of the challenge with proper interpretation of the results.

The authors state, "In our multivariate analysis" (138), but what type of multivariate analysis precisely? A few more details here would be quite useful. It would also be useful to put some statistics in text or at least a table with all the relevant values. Similarly, the authors state that the effect sizes depend on exposure to communism, but the min/max values here would be useful to know in text and also help with interpreting the data. More details are needed all around.

Thank you for this comment, we have revised the description of the methods in the Identification Strategy section (page 7) to make it clearer. We have also improved the presentation of the mathematical equation representing the estimation models: we run two separate OLS estimations for the trust in vaccines' efficiency and safety, using either dichotomous or continuous measure of exposure to communism as the explanatory variable, controlling for fixed effects of birth year, country of birth as well as for the country-specific time trends. In addition, we control for gender and a number of other controls in the robustness tests. Specifically, we add to the set of controls sequentially: dummy for living in the urban of rural area, dummy for having children, dummy for individual religiosity, and education level (primary, secondary, tertiary). On top of that, we have added the interaction term of the treatment variable (exposure to Soviet communism) with Orthodox country, as suggested above.

The table S1 with descriptive statistics of variables used in the empirical analysis was slightly reorganized and provides details concerned with: the mean, minimum and maximum values, as well as the standard deviation and the number of non-missing observations.

In the revised paper we pay more attention to the quantitative interpretation of result.

Table 1 is used to suggest that Soviet countries have lower vaccine efficiency and safety, but the countries are sorted by alphabetical order rather communism, so this pattern is difficult to see in the table. I'd recommend breaking it down with Soviet countries on one side and non-Soviet on the other so that the comparisons are much more apparent. Some descriptive statistics would also help here (Ms, SDs, etc.). The authors seem to have gone for a minimalistic presentation of the data, but the lack of details raises more questions that might easily be answered by including more details in text, in tables, etc.

Thanks for this point, we have decided to present these data graphically, using maps (Fig 1) available in public domain. Thanks to that, the difference between countries from the Soviet bloc and the rest of the world can be spotted much easier.
