# Supplementary material for: The institutional origins of vaccines distrust: Evidence from former-Soviet countries
Source: PLoS One. 2023 Mar 1;18(3):e0282420. doi: 10.1371/journal.pone.0282420 (PMC9977043; doi:10.1371/journal.pone.0282420)
Supplement: Attachment — Submitted filename: Response to review.pdf [file pone.0282420.s010.pdf]

Dear Editor,

Thank you for quick response to our resubmission.

Please find our response to your comments

- Your estimation equation suggests that year of birth fixed effects vary over time. That is not correct. These are individual specific attributes that do not vary over time. Please adjust the index in the equation - unless I am misinterpreting what you are doing.

Thank you very much for spotting this mistake, we have corrected the equation in the revised manuscript.

- Figure 1: I assume that the color-categories are based on quartiles or quintiles? Please provide the information what logic the color coding is based on. Also, it does not seem to make sense that the white category refers to values from 0 to 0!? Are these missing values? Please check and correct if necessary. (I assume this must be a mistake).

Thanks, we have refined Figure 1. Specifically, we explained the categories depicted, cleaned up the brackets in the legend, and explained that white polygons on the map represent areas with no data available. We show the values of trust in efficiency and safety of vaccines by quantile, as described in the legend to each map.

- Figure 5: Please use only standard-English terms to label your figures. You can also drop the labels on the vertical axes to be consistent.

Thanks, we have revised labels for the examined sub-groups of communist countries accordingly.

- All figures and tables (including supplementary material): I would strongly suggest to consistently use the same number of digits after the decimal point - unless there are reasons to deviate. Anything else looks very unprofessional. Also, showing about 8 digits after the decimal point does not convey any meaningful or comprehensible information, so I would suggest to limit the number of digits to a reasonable amount while still being sufficiently precise.

We appreciate this comment and agree, it was important to improve our Tables (and Figures), We use 2 decimal points in the main body of the paper, and decided to be more detailed (3 decimal points) in the Supplementary Information.

- You may also give the manuscript another read for grammar and other language mistakes. If you make any other changes to the manuscript, these should, however, be communicated.

We have revised the language and grammar very carefully, correcting text in a number of places without introducing any major changes to the contents of the paper.
